# Bivalent aptamer assemblies: A versatile toolkit for precision targeting of B-cell malignancies

**DOI:** 10.1016/j.omtn.2026.102842

**Published:** 2026-02-04

**Authors:** Ling Yin

**Affiliations:** 1 Weill Cornell Medicine, Cornell University, New York, NY, USA

## Main text

The emergence of bivalent aptamers as functional biomimetics of monoclonal antibodies marks a significant stride in the development of targeted molecular therapies. In a recent study published in *Molecular Therapy Nucleic Acids*, Cen and colleagues systematically engineered and characterized bivalent aptamer assemblies targeting CD19 and CD20—two pivotal surface antigens in B cell malignancies.^1^ By leveraging a ligand-guided selection (LIGS) platform and optimizing polyethylene glycol (PEG) linker length, the authors demonstrated that these synthetic oligonucleotide dimers not only achieve enhanced binding avidity but also replicate the CD21-dependent internalization behavior of therapeutic antibodies. This work positions bivalent aptamers not merely as binding reagents but as modular, internalizing delivery platforms, offering a promising alternative to conventional antibody-based modalities for cancers such as diffuse large B cell lymphoma (DLBCL).

B cell malignancies, including DLBCL, remain clinically challenging despite advances in immunotherapy. While monoclonal antibodies like rituximab (anti-CD20) and antibody-drug conjugates targeting CD19 have improved outcomes, their production complexity, immunogenicity, and cost limit broader application.^2^^,^^3^ Aptamers—single-stranded DNA or RNA molecules that adopt specific three-dimensional structures—present a compelling alternative due to their synthetic nature, ease of modification, and low immunogenicity.^4^ However, monomeric aptamers often exhibit modest affinity and lack the avidity-driven effects necessary for robust cell targeting and internalization. Dimerization or multimerization has thus emerged as a key strategy to enhance functional performance, with linker design playing a critical role in maintaining spatial flexibility and cooperative binding.^5^

Cen et al. first generated truncated variants of previously identified anti-CD19 and anti-CD20 aptamers to define minimal functional sequences. While truncation preserved specificity, it reduced binding affinity, prompting the authors to pursue dimerization. Through rational variation of PEG-based spacer units, they identified an optimal linker length of approximately 3.96 nm (three spacer units), which maximized apparent binding affinity at physiological temperature. Importantly, this optimized architecture also conferred temperature stability—a known hurdle for aptamers selected under non-physiological conditions. The study highlights a central principle in bivalent aptamer design: the linker must be sufficiently long to allow simultaneous engagement of two target epitopes, yet sufficiently constrained to maintain avidity and prevent intramolecular interference.

One of the most biologically insightful findings is the CD21-dependent internalization profile of the dimeric anti-CD19 aptamers. The authors show that internalization occurs efficiently in CD21-negative cell lines (Ramos, OCI-LY7, HBL-1) but is markedly inhibited in CD21-positive lines (Raji, Toledo). This mirrors the behavior of the anti-CD19 monoclonal antibody B4, as previously reported,^6^ and suggests that aptamers can faithfully recapitulate not only binding but also the intracellular trafficking pathways of their protein counterparts. From a translational perspective, this internalization specificity is clinically pertinent: approximately 30% of DLBCL cases exhibit low or absent CD21 expression,^6^ which could enable selective aptamer-mediated drug delivery to this patient subset while sparing CD21-positive normal B cells.

Equally notable is the functional profile of the dimeric anti-CD20 aptamer, which showed enhanced binding without eliciting calcium flux—a signaling response associated with certain anti-CD20 antibodies that can contribute to adverse effects.^3^ This “silent” binding characteristic may be advantageous for diagnostic imaging or drug delivery applications where receptor activation is undesirable.

Despite these advances, several technical and translational questions remain. The mass spectrometric characterization of these large, PEG-modified oligonucleotides proved challenging, with several samples showing fragmentation or adduct formation. While gel electrophoresis confirmed overall integrity, future work would benefit from orthogonal analytical approaches to ensure batch-to-batch consistency and *in vivo* stability. Moreover, the internalization assays relied on proteinase K digestion to remove surface-bound aptamers; complementary methods such as fluorescence quenching or confocal live-cell imaging could further validate the uptake kinetics and intracellular fate.

Looking forward, the modularity of aptamer architecture invites multifunctional engineering. The bivalent assemblies described here could serve as scaffolds for conjugating therapeutic payloads—small interfering RNAs (siRNAs), chemotherapy agents, or radionuclides—enabling combinatorial regimens with tunable targeting and release kinetics. Because both the targeting moiety and potential nucleic acid-based cargo share similar chemical properties, manufacturing and purification could be streamlined relative to antibody-drug conjugates. Furthermore, the ability to fine-tune linker length and valency offers a degree of control that is often more challenging to achieve with proteins.

In conclusion, Cen et al. provide a robust engineering framework for developing functional bivalent aptamers against clinically relevant cell-surface targets.^1^ Their work moves beyond proof-of-concept binding studies to demonstrate that aptamers can emulate critical biological behaviors of antibodies, including co-receptor-modulated internalization. As the field of nucleic acid therapeutics continues to mature,^7^ such tailored aptamer toolkits hold considerable promise for realizing cost-effective, stable, and precise targeted therapies for B cell malignancies and beyond.

## Declaration of interests

The author declares no competing interests.
